# Deep neural network ensemble for on-the-fly quality control-driven segmentation of cardiac MRI T1 mapping

**DOI:** 10.1016/j.media.2021.102029

**Published:** 2021-07

**Authors:** Evan Hann, Iulia A. Popescu, Qiang Zhang, Ricardo A. Gonzales, Ahmet Barutçu, Stefan Neubauer, Vanessa M. Ferreira, Stefan K. Piechnik

**Affiliations:** a Oxford University Centre for Clinical Magnetic Resonance Research (OCMR), Level 0, John Radcliffe Hospital, Headington, Oxford OX3 9DU, United Kingdom; b Çanakkale Onsekiz Mart University, Barbaros, 17100 Kepez/Çanakkale Merkez/Çanakkale, Turkey

**Keywords:** Image quality assessment, Segmentation, Ensemble neural network, Cardiovascular MRI, 62H35, 68U10, 62M45, 92C55

## Abstract

Recent developments in artificial intelligence have generated increasing interest to deploy automated image analysis for diagnostic imaging and large-scale clinical applications. However, inaccuracy from automated methods could lead to incorrect conclusions, diagnoses or even harm to patients. Manual inspection for potential inaccuracies is labor-intensive and time-consuming, hampering progress towards fast and accurate clinical reporting in high volumes. To promote reliable fully-automated image analysis, we propose a quality control-driven (QCD) segmentation framework. It is an ensemble of neural networks that integrate image analysis and quality control. The novelty of this framework is the selection of the most optimal segmentation based on predicted segmentation accuracy, on-the-fly. Additionally, this framework visualizes segmentation agreement to provide traceability of the quality control process. In this work, we demonstrated the utility of the framework in cardiovascular magnetic resonance T1-mapping - a quantitative technique for myocardial tissue characterization. The framework achieved near-perfect agreement with expert image analysts in estimating myocardial T1 value (r=0.987,p<.0005; mean absolute error (MAE)=11.3ms), with accurate segmentation quality prediction (Dice coefficient prediction MAE=0.0339) and classification (accuracy=0.99), and a fast average processing time of 0.39 second/image. In summary, the QCD framework can generate high-throughput automated image analysis with speed and accuracy that is highly desirable for large-scale clinical applications.

## Highlights

• Quality control-driven framework for cardiac segmentation and quality control.
• Exploiting variability within deep neural network ensemble to estimate uncertainty.
• Novel on-the-fly selection mechanism for the final optimal segmentation.
• Accurate, reliable, and fully automated analysis of T1 map with visualization.
• Highlighting a potential flaw of the Pearson correlation to evaluate quality score.

## Graphical abstract

**Figure:**
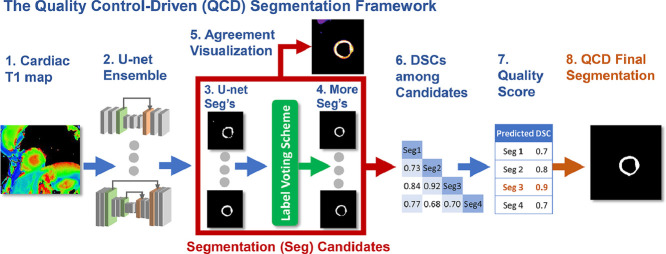

## 1 Introduction

Cardiovascular diseases (CVDs) are among the leading causes of death worldwide, killing more than 15 million people in 2016 alone (WHO, 2017). Approximately 10% (7 million) of the UK population have been diagnosed as having some form of CVD (British Heart Foundation, 2018). The high risk of mortality signifies the enormous value of tackling these diseases.

Cardiovascular magnetic resonance (CMR) is one of the major non-invasive imaging modalities for comprehensive investigation of the heart in current clinical practice. In particular, quantitative T1 mapping is an emerging CMR technique for advanced myocardial tissue characterization on a pixel-by-pixel level (Moon, Messroghli, Kellman, Piechnik, Robson, Ugander, Gatehouse, Arai, Friedrich, Neubauer, Schulz-Menger, Schelbert, 2013, Messroghli, Moon, Ferreira, Grosse-Wortmann, He, Kellman, Mascherbauer, Nezafat, Salerno, Schelbert, Taylor, Thompson, Ugander, Van Heeswijk, Friedrich, 2017), and can detect disease beyond conventional CMR methods, such as late gadolinium enhancement (LGE) imaging. T1 mapping is designated as one of the six most innovative imaging methods for evaluating patients with heart failure by the European Society of Cardiology Heart Failure Association (Čelutkiene et al., 2018). CMR T1 mapping is increasingly used in large-scale clinical studies (Petersen, Matthews, Bamberg, Bluemke, Francis, Friedrich, Leeson, Nagel, Plein, Rademakers, Young, Garratt, Peakman, Sellors, Collins, Neubauer, 2013, Kramer, Appelbaum, Desai, Desvigne-Nickens, DiMarco, Friedrich, Geller, Heckler, Ho, Jerosch-Herold, Ivey, Keleti, Kim, Kolm, Kwong, Maron, Schulz-Menger, Piechnik, Watkins, Weintraub, Wu, Neubauer, 2015) to study various cardiac diseases, including the UK Biobank imaging component (Petersen et al., 2013), which aims to scan 100,000 participants by 2021 (with >48,000 datasets acquired already).

In current practice, extraction of useful clinical parameters, such as the average myocardial T1 value, from a CMR T1 map requires manual segmentation of the left ventricular (LV) myocardium, which is a tedious, time-consuming and subjective process. In the case of the UK Biobank imaging component (Petersen et al., 2013), this could potentially require years of manual contouring for a single analyst. While sharing work between multiple analysts can speed up the process, it introduces inter-observer variability, reducing consistency, which may increase the sample size required to detect primary endpoints. Hence, there is a pressing need for processing large-scale CMR datasets consistently and efficiently. To address this need, it is desirable to develop robust, fully-automatic segmentation algorithms for advanced imaging techniques which are also reliable in quality on a per-case basis.

However, popularly-used deep learning-based automatic segmentation methods could still fail when analyzing CMR cine images, despite their overall high accuracies (Bernard et al., 2018). It is important to detect these segmentation failures automatically to avoid errors in diagnostic or research conclusions. Manual assessment of automatic segmentation quality requires visual inspection (Bai et al., 2018), at the least, and quantitative comparisons with manual contouring. The time spent on such manual quality control processes can offset the efficiency gained by automated segmentation.

### 1.1 Related work

Extensive research has been done on automating segmentation of CMR short-axis cine images for measuring LV ejection fraction (LVEF), which can potentially be adapted for T1-map processing. More than 70 segmentation algorithms, utilizing different approaches, various image-based information and statistical shape models, have been reviewed for computer-aided segmentation of CMR (Petitjean, Dacher, 2011, Peng, Lekadir, Gooya, Shao, Petersen, Frangi, 2016). Recent deep learning approaches require large training datasets, which are increasingly available with large population studies, such as the UK Biobank (Petersen et al., 2013). (Bai et al., 2018) published excellent results using a CNN-based cine MR segmentation algorithm, trained on datasets from over 4000 subjects from the UK Biobank. (Irving et al., 2017) also used deep learning for automated liver T1 map segmentation.

Ensemble deep learning segmentation models have been applied to various medical imaging applications. For example, (Zheng et al., 2019) combined 2D and 3D segmentation models with a meta-learner to segment 3D cardiac MRI data. (Kang and Gwak, 2019) combined two ResNet-based models for polyp segmentation in colonoscopy images. (Winzeck et al., 2019) used an ensemble of 5 CNNs to segment ischemic lesions in brain MRI. These studies showed that ensemble neural networks can improve segmentation accuracy. Further, the use of ensemble deep neural networks to estimate uncertainty in image classification has been proposed in (Lakshminarayanan et al., 2017). Recent research found that ensemble deep neural networks can make highly diverse predictions, compared to other state-of-the-art approaches such as Bayesian neural networks (Fort et al., 2020). Thus, it is a promising approach to estimate uncertainty. However, the application of ensemble deep neural networks for predicting segmentation quality remains unexplored.

For cardiac T1 mapping, there is limited published literature on automatic segmentation. A non-machine-learning approach was recently proposed for automatic LV segmentation and regional analysis of myocardial native T1 values (Huang et al., 2018). However, it was developed and validated only on a small cohort of healthy controls (10 subjects), which did not capture the wide range of image variability in larger databases of normal and pathological cases commonly encountered in real-life clinical practice. (Fahmy et al., 2018) proposed a fully-convolutional neural network method to segment T1 weighted images to reconstruct myocardial T1 maps. However, no mechanism of segmentation quality control for T1 mapping has been proposed.

Early research on segmentation quality control in medical imaging focused on addressing interobserver variability by deriving a reference standard from multiple manual or automatic segmentations. To estimate such reference segmentation, a simple label voting scheme can be deployed (Li et al., 2011), as well as using probabilistic schemes (Warfield, Zou, Wells, 2004, Cardoso, Leung, Modat, Keihaninejad, Cash, Barnes, Fox, Ourselin, 2013), which maximize expectation to obtain a reference segmentation. (Li et al., 2011) showed that the label voting scheme achieved better performance over probabilistic schemes in the empirical results.

Recent works of Bayesian deep learning attempted to estimate segmentation uncertainty in medical imaging. One approach is to generate multiple segmentation variants to compare variability using probabilistic neural networks (Kohl, Romera-Paredes, Meyer, De Fauw, Ledsam, Maier-Hein, Ali Eslami, Rezende, Ronneberger, 2018, Baumgartner, Tezcan, Chaitanya, Hötker, Muehlematter, Schawkat, Becker, Donati, Konukoglu, 2019) or random dropout (Roy et al., 2018). Another approach is to perform calibration when training a Bayesian neural network, such that the output probability of the voxel-wise label matches the expected accuracy (Jena and Awate, 2019). Among these studies, only (Roy et al., 2018) attempted to predict commonly-used segmentation evaluation metrics such as Dice similarity coefficient (DSC), albeit with high discrepancy. Recent research has found that the current state-of-the-art Bayesian neural networks are prone to making very similar predictions, whereas ensemble deep neural networks tend to be more diverse in making predictions (Fort et al., 2020). In other words, it is more likely for Bayesian neural networks to make similarly bad segmentation samples than for the ensemble approach. These similarly bad samples can lead to undesired overestimation of segmentation quality. In contrast, ensemble deep learning can benefit from higher prediction diversity, to achieve more robust segmentation quality control. Furthermore, the randomness inherent in the Bayesian approach with Monte Carlo sampling comes with a tradeoff on repeatability, which is an important feature for troubleshooting.

More recent work addressed segmentation quality control by predicting DSC in the absence of manual segmentation as a reference standard. As DSC is widely adopted in the image analysis research community to evaluate segmentation, it can serve as a consistent and familiar indicator of segmentation quality. For example, (Kohlberger et al., 2012) proposed to predict DSC using machine learning with handcrafted feature engineering. One limitation of this approach is the scalability of handcrafting a wider spectrum of descriptive features.

A framework based on Reverse Classification Accuracy (RCA) (Valindria et al., 2017) was introduced to predict multi-organ segmentation quality, by comparing with a database of multiple atlas-based reference segmentations. Subsequently, the RCA framework was validated using random forest-based segmentation on CMR cine images (Robinson, Valindria, Bai, Suzuki, Matthews, Page, Rueckert, Glocker, 2017, Robinson, Valindria, Bai, Oktay, Kainz, Suzuki, Sanghvi, Aung, Paiva, Zemrak, Fung, Lukaschuk, Lee, Carapella, Kim, Piechnik, Neubauer, Petersen, Page, Matthews, Rueckert, Glocker, 2019). Although the RCA framework was also validated on CNN-based segmentation, the quality prediction for CNN-based segmentation had a higher mean absolute error (MAE), compared with those for random forest-based and multi-atlas segmentations (Valindria et al., 2017). Furthermore, the RCA framework was computationally intensive, requiring 11 minutes of processing to assess the quality of a single segmentation (Robinson et al., 2019), which is not suitable for real-time clinical applications.

To support real-time clinical applications, (Robinson et al., 2018) proposed a CNN-based regression to directly map random forest-based segmentation outputs to quality control in the form of predicted DSC. However, this method was validated for random forest-based segmentation but not the popular deep learning-based segmentation.

In summary, the majority of the current automated segmentation algorithms in CMR (Bernard, Lalande, Zotti, Cervenansky, Yang, Heng, Cetin, Lekadir, Camara, Gonzalez Ballester, Sanroma, Napel, Petersen, Tziritas, Grinias, Khened, Kollerathu, Krishnamurthi, Rohe, Pennec, Sermesant, Isensee, Jager, Maier-Hein, Full, Wolf, Engelhardt, Baumgartner, Koch, Wolterink, Isgum, Jang, Hong, Patravali, Jain, Humbert, Jodoin, 2018, Bai, Sinclair, Tarroni, Oktay, Rajchl, Vaillant, Lee, Aung, Lukaschuk, Sanghvi, Zemrak, Fung, Paiva, Carapella, Kim, Suzuki, Kainz, Matthews, Petersen, Piechnik, Neubauer, Glocker, Rueckert, 2018, Petitjean, Dacher, 2011, Peng, Lekadir, Gooya, Shao, Petersen, Frangi, 2016, Irving, Hutton, Dennis, Vikal, Mavar, Kelly, Brady, 2017, Zheng, Zhang, Yang, Liang, Zhao, Wang, Chen, 2019, Kang, Gwak, 2019, Winzeck, Mocking, Bezerra, Bouts, McIntosh, Diwan, Garg, Chutinet, Kimberly, Copen, Schaefer, Ay, Singhal, Kamnitsas, Glocker, Sorensen, Wu, 2019, Huang, Huang, Chen, Wang, Huang, 2018, Fahmy, El-Rewaidy, Nezafat, Nakamori, Nezafat, 2018) do not come with segmentation quality control mechanisms suitable for automatic processing pipelines in real-life clinical applications. Moreover, quality prediction algorithms have not progressed to utilize the predicted scores to further improve segmentation accuracy.

In a proof-of-principle study, we recently proposed the quality control-driven (QCD) framework (Hann et al., 2019) to segment CMR cine images of the aorta in cross-section to estimate aortic distensibility. The QCD framework exploits the differences among multiple candidate segmentations of aortic sections, not only allowing prediction of segmentation accuracy in real-time, but also ultilizing this accuracy prediction to further improve segmentation on a per-case basis. The framework has only been validated on segmentation of simple circular aortic sections in (Hann et al., 2019) as a proof of concept. In this work, we demonstrate that the QCD framework is generalizable by applying it to left ventricular segmentation of T1-mapping images.

### 1.2 Contribution

In this work, we substantially advanced the QCD framework for automatic segmentation of CMR T1-mapping for real-time clinical applications with quality control. CMR T1-mapping is an advanced imaging technique for pixel-wise quantitative myocardial tissue characterization, and is deemed one of the 6 most innovative imaging methods for assessing patients with heart failure by the European Society of Cardiology in 2018 (Čelutkiene et al., 2018). The novel contributions of this work include the adaptability of the QCD framework to:

1. Segment a substantially different and more complex anatomical structure (the doughnut-shaped left ventricular myocardium in short-axis), compared to simple circular cross-sections of the aorta in (Hann et al., 2019). This is then generalizable to other common forms of cardiovascular imaging, such as echocardiography and cardiac computed tomography, where segmentation of the left ventricular myocardium is also commonly performed.
2. Tailor to a completely different CMR imaging protocol (quantitative mapping) from traditional cine imaging in (Hann et al., 2019), in terms of MR methodology, imaging parameters, types of artefacts, and clinical purposes.
3. Further validate improvement of segmentation accuracy on-the-fly, by selecting the most optimal LV segmentation from multiple candidates based on predicted accuracy. This concept is novel to automatic segmentation and quality control in diagnostic imaging, requiring deeper validation for various applications.
4. Include a visualization tool for segmentation agreement (novel in this work), to provide visual insights into the traditional “black-box” nature of deep-learning-based image processing, with traceability into the segmentation quality control process.
5. Additionally, we highlight a potential flaw of the Pearson correlation, commonly used as a metric for segmentation accuracy prediction. The Pearson correlation between predicted and actual observed DSCs is dependent on the performance of the segmentation method. It can be paradoxically worse for a better-performing method, and thus is not always suitable for evaluating quality prediction.

## 2 Material and methods

In this section, we first describe the origin of the data used in the development and testing of the novel quality control-driven (QCD) framework. Then, we introduce the methodology of the segmentation component of the framework, and the methodology of the automatic quality control of segmentation, with segmentation quality visualization. We also present the detailed implementation and evaluation of the QCD framework.

### 2.1 Material

The development and testing data comprised of 2383 CMR native (pre-contrast) T1 maps using the ShMOLLI T1-mapping method (Piechnik et al., 2010), zero-padded to 384×384 pixels. All T1 maps were short-axis views of the left ventricular (LV) myocardium, varying from basal to very apical slices. Endo-and epicardial contours were manually segmented as part of our prior research studies (Dall’Armellina, Piechnik, Ferreira, Si, Robson, Francis, Cuculi, Kharbanda, Banning, Choudhury, Karamitsos, Neubauer, 2012, Ferreira, Piechnik, Dallarmellina, Karamitsos, Francis, Choudhury, Friedrich, Robson, Neubauer, 2012, Dass, Suttie, Piechnik, Ferreira, Holloway, Banerjee, Mahmod, Cochlin, Karamitsos, Robson, Watkins, Neubauer, 2012, Piechnik, Ferreira, Lewandowski, Ntusi, Banerjee, Holloway, Hofman, Sado, Maestrini, White, Lazdam, Karamitsos, Moon, Neubauer, Leeson, Robson, 2013, Bull, White, Piechnik, Flett, Ferreira, Loudon, Francis, Karamitsos, Prendergast, Robson, Neubauer, Moon, Myerson, 2013, Karamitsos, Piechnik, Banypersad, Fontana, Ntusi, Ferreira, Whelan, Myerson, Robson, Hawkins, Neubauer, Moon, 2013, Ferreira, Piechnik, Dall’Armellina, Karamitsos, Francis, Ntusi, Holloway, Choudhury, Kardos, Robson, Friedrich, Neubauer, 2013, Ferreira, Piechnik, Robson, Neubauer, Karamitsos, 2014, Ntusi, Piechnik, Francis, Ferreira, Rai, Matthews, Robson, Moon, Wordsworth, Neubauer, Karamitsos, 2014, Ferreira, Piechnik, Dall’Armellina, Karamitsos, Francis, Ntusi, Holloway, Choudhury, Kardos, Robson, Friedrich, Neubauer, 2014, Mahmod, Piechnik, Levelt, Ferreira, Francis, Lewis, Pal, Dass, Ashrafian, Neubauer, Karamitsos, 2014, Ntusi, Piechnik, Francis, Ferreira, Matthews, Robson, Wordsworth, Neubauer, Karamitsos, 2015, Levelt, Mahmod, Piechnik, Ariga, Francis, Rodgers, Clarke, Sabharwal, Schneider, Karamitsos, Clarke, Rider, Neubauer, 2016, Ferreira, Wijesurendra, Liu, Greiser, Casadei, Robson, Neubauer, Piechnik, 2015, Ntusi, O’Dwyer, Dorrell, Wainwright, Piechnik, Clutton, Hancock, Ferreira, Cox, Badri, Karamitsos, Emmanuel, Clarke, Neubauer, Holloway, 2016, Ferreira, Marcelino, Piechnik, Marini, Karamitsos, Ntusi, Francis, Robson, Arnold, Mihai, Thomas, Herincs, Hassan-Smith, Greiser, Arlt, Korbonits, Karavitaki, Grossman, Wass, Neubauer, 2016). The manual contours served as the ground truth (GT) segmentations for evaluating automatic segmentations and for deriving the reference DSCs to train and test the automatic segmentation quality predictors. The data were randomly split into 80% training data, 9% validation data, and 11% testing data.

### 2.2 Multiple segmentation models

**Fig. 1:**
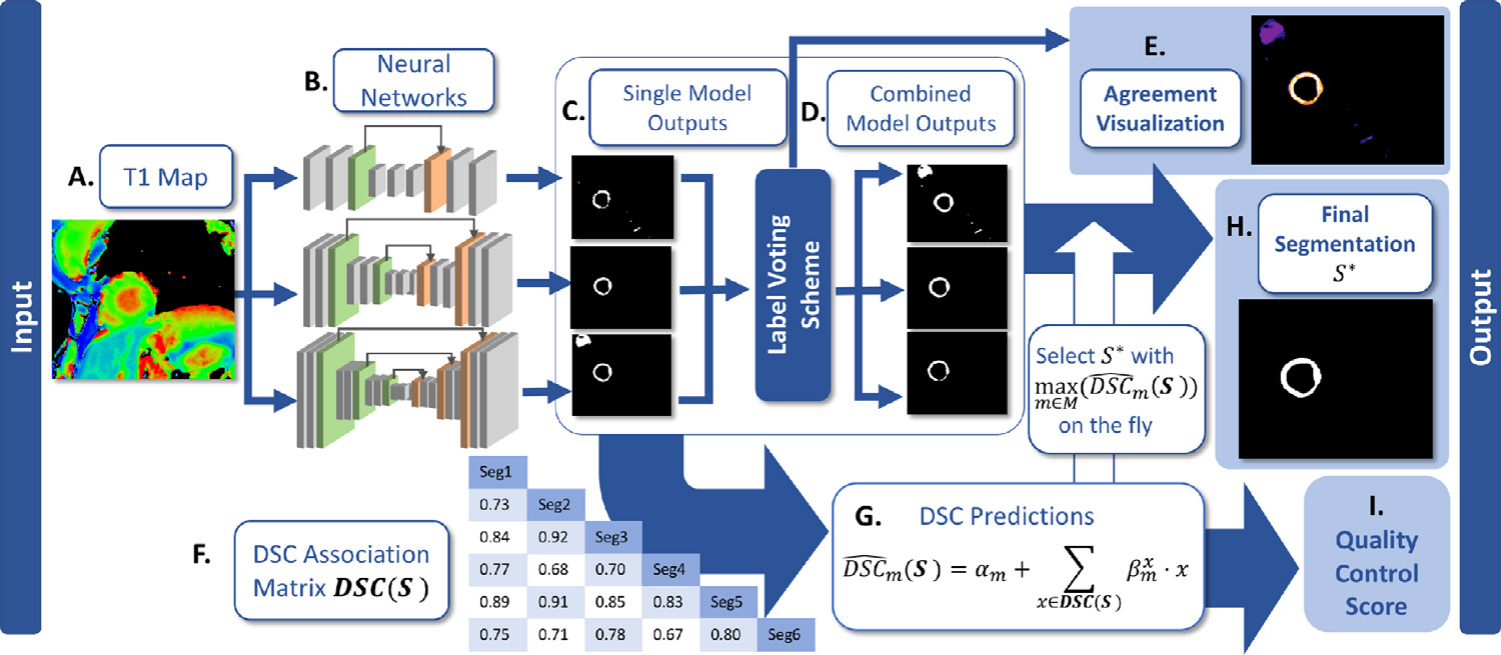
Overview of the multiple neural network framework for integrated segmentation and quality control. For simplicity, this illustration shows an example of 3 single independent neural networks. (A) A T1 map is analyzed by (B) independent segmentation models to output (C) single-model segmentations. Then, the single-model segmentations are passed to a label voting scheme to generate (D) combined-model segmentations. (E) In addition, the agreement of the segmentation can be visualized. (F) A DSC matrix is generated from both single model and combined-model segmentations for (G) DSC predictions with regression models. (H) The final segmentation is chosen based on the DSC prediction, and the corresponding predicted DSC is output as (I) the final quality control score.

The QCD framework uses multiple segmentation models, where each model m∈M generates a segmentation $S^{m}$ of an input T1 map (Fig. 1A). $S^{m}$ is a binary pixel-classification mask where the LV myocardium is labeled as 1, and other pixels as 0.

There are two types of segmentation models in the framework: single models (Fig. 1C) and combined models (Fig. 1D). For an input T1 map, each single model, such as a single convolutional neural network, can independently generate a segmentation (Fig. 1B). In this work, a range of fully convolutional neural networks of different depths, such as U-net 7, U-net 11, and so on, are used to make a diverse set of candidate segmentations. This is analogous to the spread of expertise in a multidisciplinary clinical team. Furthermore, these single model segmentations can also be combined via a label voting scheme (Li et al., 2011) to generate additional segmentation candidates, which we term combined segmentations. All available single model segmentations, denoted as **J**, of an input T1 map are summed up in a pixel-wise fashion, then thresholded by t∈{1,2,…,∣J∣} such that

(1) $$K^{t}\left(u,v\right)=\left\{ \begin{array}{cc} 1 & \text{if}\;\sum_{J\in J}J(u,v)\geq t\\ 0 & \text{otherwise,} \end{array}\right.$$

where (u,v) is a pixel coordinate in the T1 map, and $K^{t}$ denotes a combined segmentation generated with a threshold parameter t. This generates ∣J∣ (the number of neural networks used) additional segmentation variants for each input image.

### 2.3 Visualization of segmentation agreement

The agreement of the single neural network model segmentations is visualized by color-coding the pixel-wise summation map $\sum_{J\in J}J(u,v)$ in Eq. (1). It highlights the degree and location of segmentation differences among single neural network models (Fig. 1E), and unmasks the “black-box” nature of the deep learning-based segmentation, facilitating transparency of the quality control process in the framework. In addition, as combined segmentations are generated similarly by overlaying the single model segmentations pixel-by-pixel, the visualization also shows the agreement of the combined segmentations.

### 2.4 Automatic quality control of segmentation

In addition to fully-automatic segmentation, the framework is capable of generating an inherent quality score of any segmentation $S^{m}$ produced by a model m∈M, in the absence of the manual ground truth (GT) segmentation $S^{GT}$. M denotes all the available single and combined models in the framework. For any segmentation $S^{m},$ the framework predicts Dice similarity coefficient $DSC(S^{m},S^{GT})$ as the segmentation quality score (Fig. 1G).

The quality scoring exploits the differences in segmentation among all available candidate segmentation outputs to generate the quality score. The quality scoring relies on a negative relationship between the segmentation differences and the segmentation quality.

In order to establish this relationship, we quantify and compare the differences in segmentation among the multiple segmentation models implemented in M. $DSC(S^{m},S^{n})$ is computed for every pair of distinct models (m,n)∈M×M and m≠n. Hence, we obtain an association matrix of inter-segmentation Dice coefficients DSC(S) (Fig. 1E), where $S=(S^{m},S^{n},\ldots )$ represents all the available segmentations in the framework for an input T1 map.

Subsequently, for each segmentation model m∈M, a quality scoring model is needed to predict $DSC(S^{m},S^{GT})$ of any image. The Dice coefficient prediction ${\hat{DSC}}_{m}\left(S\right)$ is based on multiple linear regression, such that

(2) $${\hat{DSC}}_{m}\left(S\right)=\alpha_{m}+\sum_{x\in \mathrm{DSC}(S)}\beta_{m}^{x}\cdot x,$$

where $\alpha_{m}$ and $\beta_{m}$ are the linear regression parameters, trained individually for each segmentation model m∈M using the training data, where the ground truth manual segmentation $S^{GT}$ is available to compute $DSC(S^{m},S^{GT})$.

### 2.5 Quality control-driven segmentation

The availability of quality prediction for each candidate segmentation in the framework enables on-the-fly selection of the final segmentation from all the available segmentations. For a T1 map, the segmentation $S^{m}$ generated by a model m∈M is automatically assigned a quality score, in the form of a predicted Dice similarity coefficient ${\hat{DSC}}_{m}\left(S\right)$. Assuming that the predicted Dice coefficient (Fig. 1G) is accurate, the segmentation $S^{m}$ with a higher ${\hat{DSC}}_{m}\left(S\right)$ is expected to achieve a higher $DSC(S^{m},S^{GT})$. Hence, we select the segmentation with the highest quality score $max_{m\in M}\left({\hat{DSC}}_{m}\left(S\right)\right)$ to be the final, most optimal segmentation $S^{*},$ for each T1 map (Fig. 1H). We expect that this novel quality control-driven (QCD) approach can improve the overall segmentation accuracy.

Two additional variants of the QCD segmentation are considered in this work for comparison. The default QCD framework includes both single models and combined models as candidates. The final segmentation is selected based on the highest predicted DSC. The first variant (QCD-Lite) is similar to the default QCD framework. The only difference is that the combined models are excluded from the candidates for the QCD-Lite. This creates a “lighter” version of the default QCD framework. The same independently-trained single models from the default QCD are used as candidates in the QCD-Lite. The DSC predictors are retrained to accommodate fewer candidate models. This is a preliminary attempt to assess how the choice of candidate models impacts on the segmentation performance. Extending upon the default QCD framework, the second variant (weighted average QCD) assigns the corresponding predicted DSC as a weight to each candidate segmentation. It then outputs a weighted average segmentation as the final output, instead of selecting only one optimal segmentation. The DSC prediction for the final segmentation is also a weighted average. This is to explore the possibility of further improving the QCD framework.

### 2.6 Implementation

For the specific implementation of the QCD framework, 6 independent U-nets (Ronneberger et al., 2015) were included into Nets to perform automated LV myocardium segmentation. Each of them varied in hyper-parameters, such as the number of convolutional layers, pooling layers, and the number of skip connections. The smallest neural network implemented had only 7 convolutional and transposed convolutional layers, and 1 skip connection, while the deepest neural network had 27 layers and 6 skip connections. We refer to each of the neural networks by the number of convolutional and transposed convolutional layers as follows: U-net 7, U-net 11, and so forth, up to U-net 27. The wide range in capacity of the networks is intentional to introduce more diverse variation in segmentation. The neural networks were independently trained, using the Adam optimizer (Kingma and Ba, 2014) to minimize the cross-entropy loss in the training data of CMR T1 maps. The framework was trained and validated on a single desktop computer using a single NVIDIA Titan X GPU, with 12GB onboard memory and 3072 cores. Each convolutional neural network of the ensemble was independently trained for 60 epochs.

### 2.7 Evaluation methods

For each model m∈M, the segmentation performance was evaluated by averaging $DSC(S^{m},S^{GT})$ between the automated segmentation $S^{m}$ and the manual segmentation $S^{GT}$ of T1 maps in the validation data.

The accuracy of the DSC prediction was also evaluated using the validation data by mean absolute error (MAE) and Pearson correlation coefficient (r) of the prediction ${\hat{DSC}}_{m}S$ and the prediction target $DSC(S^{m},S^{GT})$ for each model m∈M.

The DSC prediction was further evaluated for binary classification of good (observed DSC ≥0.7) and poor (observed DSC <0.7) segmentation. The threshold of 0.7 was chosen based on (Robinson et al., 2019). The binary classification was evaluated according to the accuracy (TP+TN)/(TP+FP+TN+FN), the true positive rate TP/(TP+FN), and the false positive rate FP/(FP+TN), where TP, FP, TN, and FN respectively denote the number of true positive cases (observed DSC ≥0.7 and predicted DSC ≥0.7), false positive cases (observed DSC <0.7 and predicted DSC ≥0.7), true negative cases (observed DSC <0.7 and predicted DSC <0.7), and false negative cases (observed DSC ≥0.7 and predicted DSC <0.7). The binary classification can further demonstrate the practical usage of the DSC prediction in the QCD framework.

The estimated myocardial T1 value, calculated by averaging the T1 values of all pixels in the myocardium, was identified by the automated method, for each T1 map in the testing data. Similarly, we established the ground truth T1 value using the manual segmentation. The T1 estimation was evaluated using mean error, mean absolute error (MAE), and Pearson correlation (r) between the estimated values and the ground truth. In addition, the relative errors of T1 were categorized by manual image quality assessments by a consultant cardiologist (AB), who classified the T1 maps into 4 levels of quality: ‘excellent’, ‘good’, ‘acceptable’, and ‘poor’ (Table 1).

**Table 1:** Image quality categories for T1 maps described by expert human operators.

Category	Description	Proportion
Excellent	Well-defined borders of the myocardium with good contrast. Typically, mid-ventricular slice. Easy to contour with high consistency.	5.2%
Good	Overall well-defined borders of the myocardium with reasonably good contrast. Requires some caution when contouring. Moderately easy to contour, but prone to higher variability than easy cases.	23.5%
Acceptable	Ambiguous borders of the myocardium with poor contrast. Requires caution when contouring. Prone to high variability.	65.3%
Poor	Ambiguous borders of the myocardium with poor contrast. Observable pathologies or artefacts.	6.0%

To demonstrate generalizability, the QCD segmentation framework was trained and tested on the Sunnybrook cardiac dataset (Radau et al., 2009), for a seperate application. The evaluation results are presented in Appendix D.

## 3 Results

The neural networks and the DSC predictors were trained on 1906 CMR T1 maps, and were subsequently evaluated on previously unseen validation data of 220 T1 maps. With a single GPU, the framework took 15 minutes and 21 seconds (including data I/O time) to segment the entire dataset of 2383 T1-maps and produce the quality control scores. On average, one image took 0.39 second to process.

### 3.1 Accuracy of segmentation

Among the 12 individual segmentation models investigated for the QCD framework, Combined Model 3 had the highest mean observed DSC of 0.8371 (Table 2), followed closely by Combined Model 2 (DSC=0.8368), both outperforming the deepest single neural network U-net 27 (DSC=0.8313).

**Table 2:** Segmentation performance evaluated in mean Dice similarity coefficient (DSC) and standard deviation (SD) with manual segmentation as the ground truth, and DSC prediction performance evaluated in mean absolute error (MAE) and Pearson correlation (r). All r had p <0.0005.

Segmentation Model	Mean DSC (SD)	MAE	***r***
U-net 7	0.6688 (0.1715)	0.0312	0.95
U-net 11	0.8091 (0.0738)	0.0368	0.73
U-net 15	0.8301 (0.0678)	0.0380	0.66
U-net 19	0.8264 (0.0547)	0.0384	0.43
U-net 23	0.8309 (0.0556)	0.0382	0.57
U-net 27	0.8313 (0.0578)	0.0399	0.42
Combined Model 1	0.7896 (0.0769)	0.0463	0.60
Combined Model 2	0.8368 (0.0598)	0.0405	0.47
Combined Model 3	0.8371 (0.0565)	0.0382	0.52
Combined Model 4	0.8288 (0.0576)	0.0354	0.73
Combined Model 5	0.8087 (0.0725)	0.0333	0.88
Combined Model 6	0.6883 (0.1779)	0.0335	0.96
QCD	0.8508 (0.0541)	0.0339	0.53
QCD-Lite	0.8503 (0.0562)	0.0344	0.58
Weighted Average QCD	0.8225 (0.0590)	0.0315	0.71

**Fig. 2:**
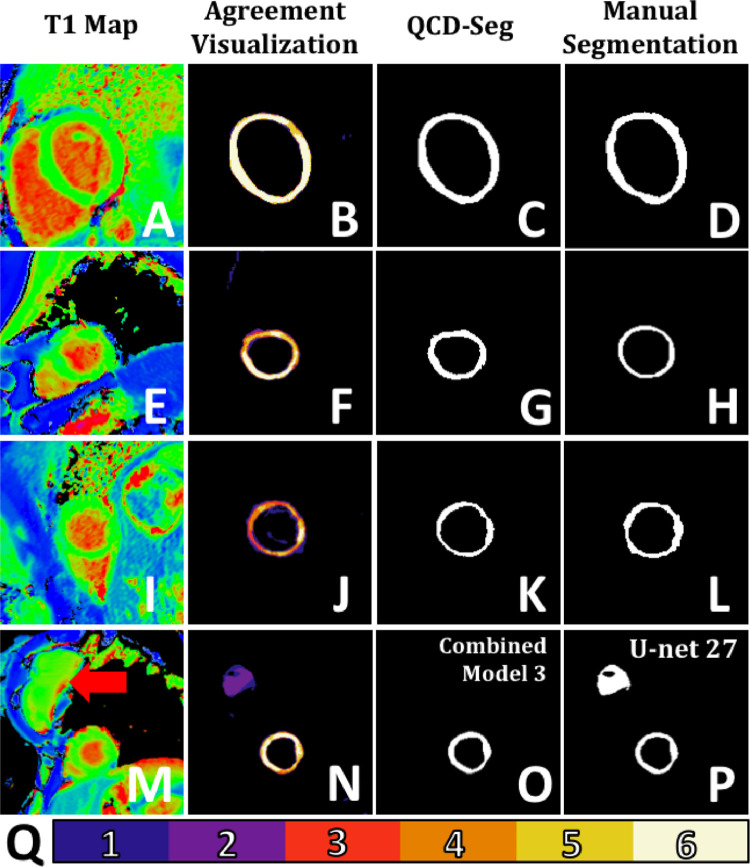
Examples of T1 maps, agreement visualizations, and segmentations. (A-D) The top row is an example in which there was high agreement among segmentation models, as shown in (B) the agreement visualization. Hence, the predicted DSC of the QCD output (C) was high (0.8933), which was consistent with the DSC (0.8996). (E-H) The second row is an example in which there was some disagreement among the segmentation models, as shown in (F) the agreement visualization. Hence, the predicted DSC of the QCD output (G) was low (0.6550), which was consistent with the DSC (0.6425). (I-L) The third row is an example in which the agreement visualization (J) showed high disagreement among the segmentation models, possibly due to the heavy wraparound artefact. The predicted DSC was low (0.5404) due to the disagreement despite that the DSC was much higher (0.7912). In clinical practice, this T1 map (I) should be treated with caution. Thus, a lower predicted DSC can serve as a useful alert. (M-P) The last row shows an example in which (P) the deepest single neural network (U-net 27) falsely classified the breast implant (red arrow in M) as part of the myocardium. On the other hand, (O) Combined Model 3 produced more robust segmentation. (Q) is a color bar which indicates the degree of agreement in the visualizations, with 1 being the lowest agreement to 6 being the highest agreement. (For interpretation of the references to colour in this figure legend, the reader is referred to the web version of this article.)

Pictorial examples of the T1 maps and their corresponding segmentations can be seen in Fig. 2. Specifically, Fig. 2M-P shows an example that Combined Model 3 generated more robust segmentation than U-net 27. In this case, U-net 27 misclassified the breast implant (indicated by a red arrow in Fig. 2M) as the myocardium. This case demonstrated the advantage of the on-the-fly selection of the final segmentation combined with the label voting approach, instead of using a fixed segmentation model or a fixed weighted-average segmentation.

**Fig. 3:**
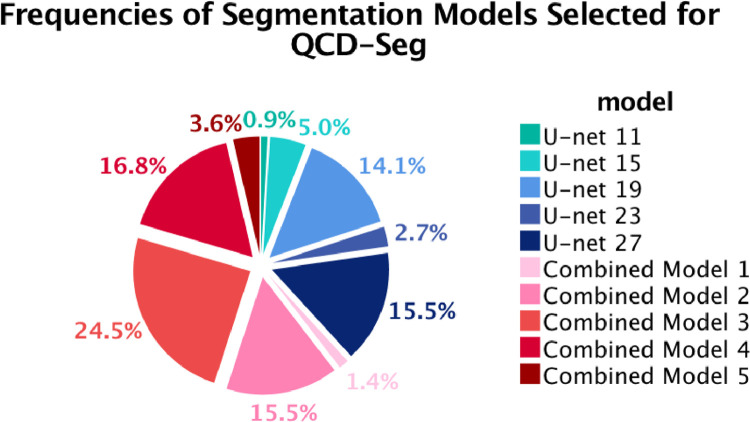
Pie chart of frequencies of the segmentation models selected for the final segmentation in the QCD framework. It shows that outputs generated by Combined Models 2, 3, 4 were most frequently selected as the optimal segmentations, accounting for more than half of the cases in the validation data. No segmentation generated by U-net 7 or Combined Model 6 was selected by the QCD framework.

The QCD framework and the QCD-Lite variant further outperformed any individual segmentation models and demonstrated the best performance in the LV myocardium segmentation on the validation data, with a DSC value of 0.8508 and 0.8503, respectively (Table 2). The QCD framework and the QCD-Lite also outperformed the weighted average QCD variant, which obtained a DSC of 0.8225. This demonstrated the effectiveness of the optimal segmentation model selection with the highest predicted quality obtained on-the-fly. This is similar to our clinical experience that averaging may fall short when multiple human analysts have different training and experiences. The segmentations produced may not form linear relationships. Combined Models 2, 3 and 4 contributed the most to the QCD segmentation, accounting for more than half of the final segmentation outputs (Fig. 3).

### 3.2 Visualization of segmentation agreement

The agreement visualization of segmentation shows a spatial map of agreement among the multiple single neural networks. Additional examples of the agreement visualization can be seen in Fig. 2. Fig. 2Q is the color bar of scale from 1 to 6, indicating the number of single neural networks which identify a particular pixel as the myocardium, hence showing the extent of agreement among the neural networks. Fig. 2B shows an agreement visualization with generally high degree of segmentation agreement across the myocardium segmentation. Thus, the automated segmentation (Fig. 2C) was also expected to highly agree with the manual segmentation (Fig. 2D). Fig. 2F shows that the neural networks disagreed with each other mostly at the apical anterior wall. This is the same region where the automated segmentation (Fig. 2G) differed from the manual segmentation (Fig. 2H). Fig. 2J shows generally high disagreement among the neural networks across the myocardium, possibly due to the heavy wraparound artefact in the T1 map (Fig. 2I). Thus, a low predicted DSC was expected. Fig. 2N shows a high disagreement at the breast implant (purple-colored pixels). These examples show that the agreement visualization can highlight the regions where disagreements happen and provide insights into the quality control of the segmentation process.

### 3.3 Accuracy of segmentation quality control

The MAEs for the DSC prediction ranged from 0.0312 to 0.0463, for all implemented models (Table 2), indicating overall good prediction of quality control for all the candidate segmentations, substantiating the validity of the QCD framework. The MAE in predicting the DSCs for the QCD framework was 0.0339 (Table 2). Multiple linear regression coefficients for the DSC prediction of each candidate segmentation model are provided in Table A.1.

**Table 3:** Agreement of the estimated T1 values using the automated QCD segmentation compared with manual segmentation in the testing data.

Pearson Correlation	0.987	(p<.0005)
**Mean Error (SD)**	-4.6ms	(16.7)
**Mean Absolute Error (SD)**	11.3ms	(13.0)

**Fig. 4:**
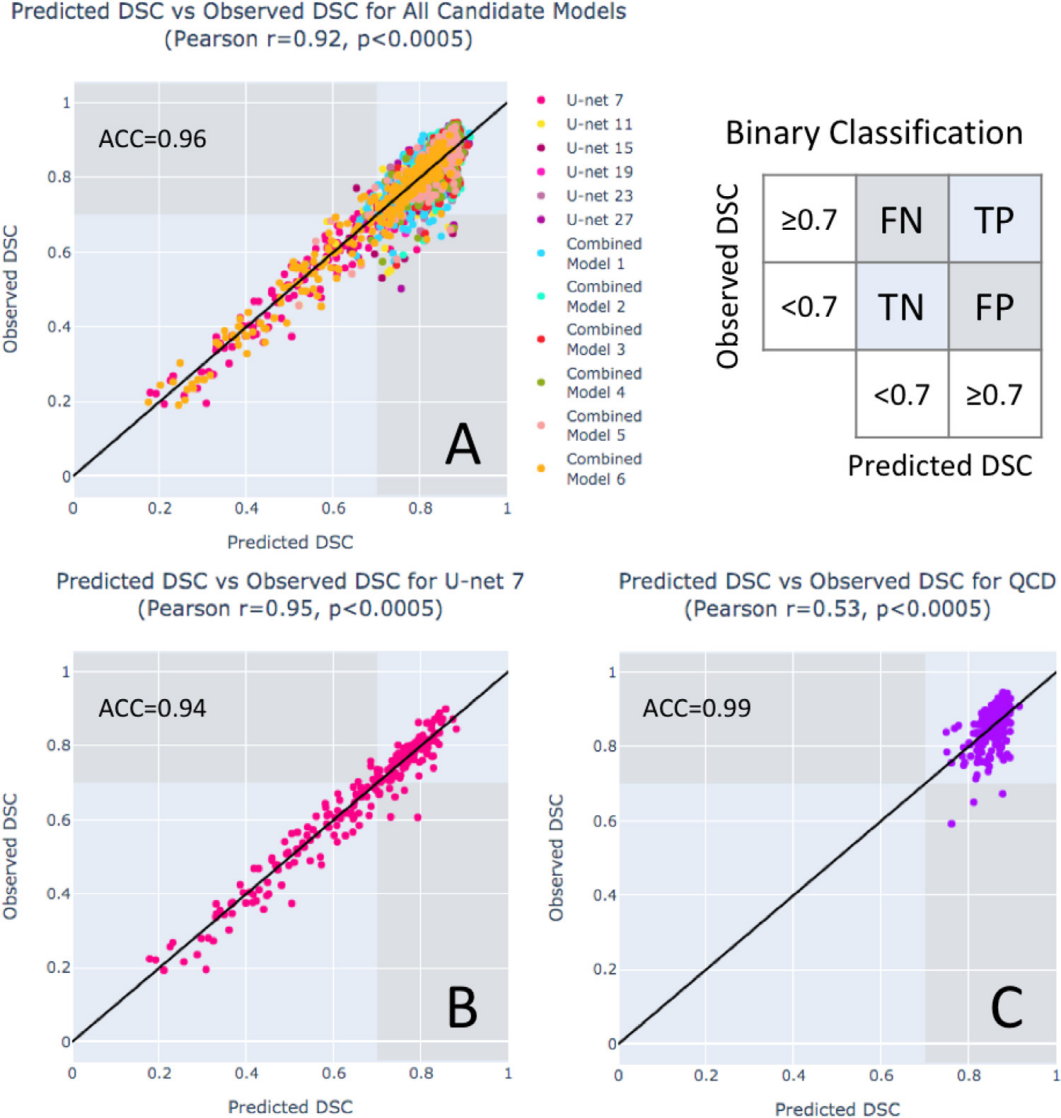
**The Pearson correlation coefficient is not necessarily an accurate indicator of quality prediction performance.** Scatter plots of the predicted vs the observed DSCs are shown for: (A) all the candidate segmentations in the QCD framework, (B) U-net 7, and (C) the final segmentations selected by the QCD framework. The highest classification accuracy (ACC) of good (observed DSC ≥0.7) and bad (observed DSC <0.7) segmentations is seen in (C) the final segmentations selected by the QCD framework (ACC=0.99), compared to (A) all the candidate segmentations (ACC=0.96) and (B) U-net 7 (ACC=0.94). Although high correlations were observed for (A) all the candidate segmentations (r=0.92) and (B) U-net 7 (r=0.94), a much weaker correlation was obtained for (C) the final QCD segmentations (r=0.53), which had a better segmentation performance (observed DSC between 0.59-0.95) and despite having the highest accuracy (ACC=0.99).

The Pearson correlation of the predicted DSCs and the observed DSCs was calculated for each model (Table 2), and is often used to assess the performance of segmentation quality control methods. Fig. 4A shows high correlation (r=0.92,p<.0005) for the DSC prediction of all the candidate segmentations. This indicates that the DSC prediction can estimate a wide range of segmentation quality for all the candidate segmentations. Interestingly, the correlations measured individually for the segmentation models (Table 2) show that the Pearson correlation tended to be stronger if the segmentation model performed worse in terms of mean DSC, and, conversely, weaker if the segmentation model performed better. Fig. 4B and C explain the relation using the scatter plots of the predicted DSCs and the observed DSCs for U-net 7 and the QCD final segmentations, respectively. For the shallowest U-net 7, a strong linear correlation (r=0.95,p<.0005) can be clearly observed as the data points spread along the identity line from 0.19 to 0.90 (Fig. 4B). However, for the QCD final segmentations, the Pearson correlation (r=0.53,p<.0005) of quality control was weak (Table 2) despite the high mean DSC and the low MAE in the DSC prediction, as the data points in the scatter plot cluster around 0.59 to 0.95 (Fig. 4C). Therefore, the Pearson correlation is not necessarily a good metric for evaluating the quality control component in this work, and may be misleading when the accuracy of the segmentation models is very high.

The DSC prediction in the QCD framework was further evaluated for binary classification of good (observed DSC ≥0.7) and bad (observed DSC <0.7) segmentations. The evaluation showed high classification accuracy (ACC) for all the candidate segmentations (ACC=0.96, Fig. 4A), U-net 7 (ACC=0.94, Fig. 4B), and the final segmentations selected by the QCD framework (ACC=0.99, Fig. 4C). High true positive rates (TPR) were also achieved: 0.99 for all the candidate segmentations, 0.94 for U-net 7, and 1.00 for the QCD final segmentations. In addition, the false positive rates (FPR) were reported: 0.25 for all the candidate segmentations, and 0.04 for U-net 7. Only 3 false positive cases, with high predicted DSCs (≥0.7) but low observed DSCs (<0.7), were found for the 220 QCD final segmentations. These results demonstrated that the DSC prediction can differentiate good and poor segmentations for quality control purpose.

**Fig. C.1:**
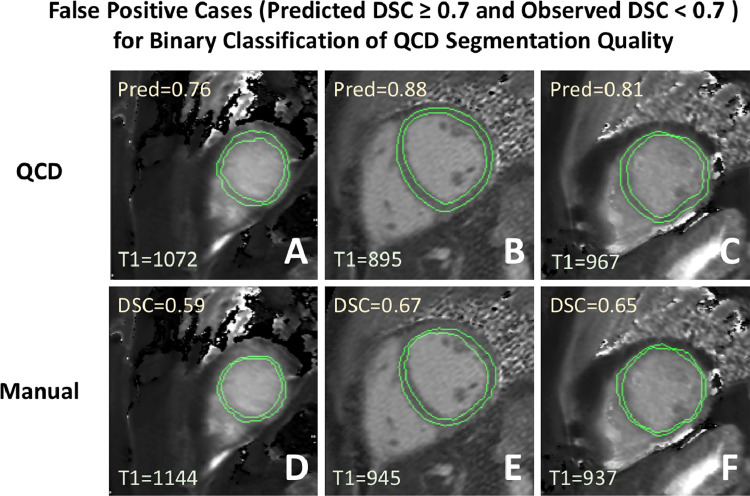
False positive cases (high predicted DSC but low observed DSC) for binary classification of the QCD final segmentation quality. The segmentations in the top row (A, B, C) were output by the QCD framework. These contours appeared acceptable and had high predicted DSCs (≥0.7). The bottom row (D, E, F) shows the corresponding manual contours, which appeared excessively eroded by the human operator, a valid approach in some studies aiming to limit partial volume effects. In these cases, the observed DSC values were “unfairly” low due to the low overlap between the narrow manual myocardial segmentations and the corresponding QCD outputs. Despite the low DSCs, the myocardial T1 values estimated by the QCD segmentations agreed with the manual estimations to within ±6.5%.

The 3 false positive cases for the QCD segmentations were identified (Fig. C.1). The automatic segmentations (Fig. C.1A-C) for these cases appeared acceptable after review for practical use despite having low observed DSCs. The manual segmentation masks (Fig. C.1D-F) were excessively thin, potentially due to attempts by the human operator to avoid partial volume when myocardial coverage was not considered critical (Piechnik et al., 2013; ?). This contributed to the low observed DSCs due to little overlap between the automatic segmentations and the thin manual masks. Despite the low DSCs, the myocardial T1 values estimated by the QCD agreed with the manual estimation to within ±6.5%.

### 3.4 T1 value estimation

**Fig. 5:**
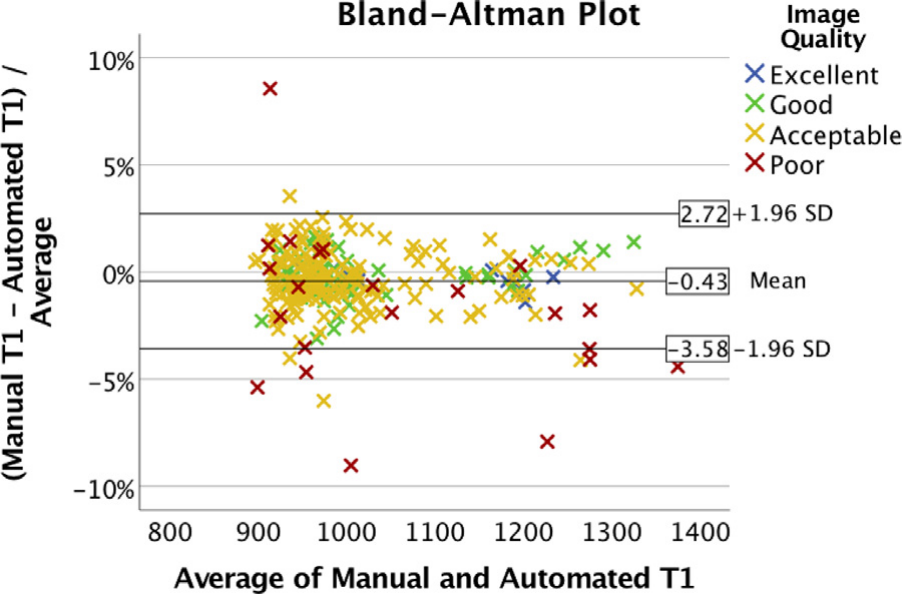
Bland-Altman plot of agreement between T1 values estimated using automated and manual segmentations. Different colors indicate the image quality as perceived by the expert human operator. Most of the points were in the range of -3.58% to 2.72% difference. Cases outside of this range were “poor-quality” (7 cases) and “acceptable” (4 cases).

The QCD achieved the highest mean DSC (Table 2), and thus was chosen for estimating the LV myocardium T1 values in the testing data. The result showed a high degree of agreement for the estimated T1 values between manual and automatic segmentations, with a mean error of -4.6ms, a mean absolute error (MAE) of 11.3ms, and a Pearson correlation r=0.987 (p<.0005, Fig. 3). The Bland-Altman plot (Fig. 5) showed consistent estimation of the T1 values, with a 95% confidence interval (CI) from -3.58% to 2.72% for the differences between the automatic and the manual segmentations. There was no apparent correlation between the T1 estimation error and the average T1, indicating that the error was not dependent on the T1 value.

Further investigation found 11 outlier cases outside the 95% CI range in the Bland-Altman plot (Fig. 5), where 7 cases were classified as ‘poor’ image quality, and 4 were ‘acceptable’.

## 4 Discussion

The novel real-time quality control-driven (QCD) approach was successfully applied to CMR T1 mapping automated image segmentation, with speed, accuracy, reliability, and visualization for the purposes of real-world diagnostic medical imaging. This is demonstrated by the use of the per-case DSC prediction to select the most optimal segmentation on-the-fly from multiple intermediate candidates. This QCD framework achieved high agreement in myocardial T1 values between the automated and the manual segmentations. Furthermore, the fast processing speed of 0.39s/image enables real-time clinical applications. In addition, the analysis of the Pearson correlation and the segmentation performance exposed an undesirable dependence between the two, showing that the Pearson correlation may not always be a suitable evaluation metric for quality prediction.

### 4.1 Comparisons with related work

The QCD framework demonstrated high accuracy in estimating LV myocardial mean T1 value in CMR images. This framework showed high consistency with the manual estimation of the myocardial T1 value, compared to the inter-observer variability between two human operators using the same T1-mapping method in (Dass et al., 2012), which reported a Pearson correlation of 0.92 with the 95% CI of relative errors ranging from -4.7% to 3.3%. The QCD framework also showed a higher Pearson correlation of T1 estimation than that reported by (Fahmy et al., 2018) (r=0.72,p<.0001). (Huang et al., 2018) reported a small error in estimating T1 values, with the mean relative absolute error of 4.6%. However, only 10 healthy subjects were studied in their work, which may not reflect the adaptability of their method to the real-world clinical setting where a wide range of pathologies exist.

The QCD framework demonstrated high accuracy in quality control by predicting the DSC of the segmentation, regardless of the availability of manual segmentation as the ground truth. We identified existing CMR image segmentation quality control frameworks for comparison, though it is important to note that the training and testing data used were different. We achieved low MAE (0.0339) compared with the RCA quality control frameworks (Valindria, Lavdas, Bai, Kamnitsas, Aboagye, Rockall, Rueckert, Glocker, 2017, Robinson, Valindria, Bai, Suzuki, Matthews, Page, Rueckert, Glocker, 2017, Robinson, Valindria, Bai, Oktay, Kainz, Suzuki, Sanghvi, Aung, Paiva, Zemrak, Fung, Lukaschuk, Lee, Carapella, Kim, Piechnik, Neubauer, Petersen, Page, Matthews, Rueckert, Glocker, 2019), in which the reported prediction MAE was at least 0.044. A CNN-regression approach (Robinson et al., 2018) also reported a higher MAE (0.055) in predicting the segmentation of the LV myocardium. The low MAE of the DSC prediction achieved by the QCD framework also compares favorably with the dropout-based quality control method (Roy et al., 2018), which appeared to have a high discrepancy in predicting DSC. Unlike the QCD framework, the dropout-based approach does not have the advantage to utilize regression for more accurate DSC prediction, due to the randomness inherent to this approach.

The binary classification of good (observed DSC ≥0.7) and poor (observed DSC <0.7) segmentations demonstrated high classification accuracy of 0.96 for all the candidate segmentations and 0.99 for the final segmentations in the QCD framework. This is on par with the results (classification accuracy of over 0.95) reported by (Robinson et al., 2019).

The whole framework (both the segmentation and the quality control) is faster (0.39 s/image) than the RCA framework, which required 11 minutes to process a single image (Robinson et al., 2019). Expectedly, the QCD framework, which utilized 6 fully convolutional neural networks, was slower than the single CNN used in (Robinson et al., 2018), but only by a small fraction of a second. This demonstrates that the fast processing speed of the QCD framework permits real-time clinical applications.

### 4.2 Limitations and future work

The single final segmentation selection mechanism in the QCD framework is flexible to include different segmentation methods, and techniques to combine segmentations. Further research can be done to assess potential benefits of incorporating a more diverse variety of segmentation methods such as active contour models (Kass et al., 1988), or multi-atlas segmentation (Iglesias and Sabuncu, 2015). The use of different segmentation algorithms can potentially further strengthen the reliability of the segmentation and the quality control of the framework by imposing anatomical constraints used in active contour models or multi-atlas segmentation. Furthermore, future research can investigate the inclusion of different techniques to combine single model segmentations, such as by weighted averaging, as candidates to be chosen as the final output in the QCD framework. With ever-advancing research in medical image analysis, one of the strongest points of this framework is that it can incorporate any prior and future classification models as intermediate solutions, which may further improve both accuracy and reliability of the overall classification process. In addition, research on better selection and choice of candidate segmentation algorithms can be beneficial in further optimization of the QCD framework.

In this work, we focused on the quality control of automated segmentation, as a first step towards clinical translation of automated image post-processing. In the future, we aim to adapt the presented quality control-driven framework to ensure reliability of the extraction of clinical parameters from multimodal data.

The performance comparisons of the segmentation and the quality control methods between various publications need to be treated with care due to potentially significant differences of the datasets. The work presented is a proof-of-principle of the QCD framework, derived using internal datasets; further training and validation, including head-to-head comparisons of segmentation and quality control performance, using large-scale external datasets, such as the UK Biobank (Petersen et al., 2013), will be beneficial for wider generalizability, and is future work in the pipeline.

Further work is required to address in detail any potential challenges, i.e. data shift, or validating the QCD framework on a variety of imaging modalities using large-scale external datasets, such as the UK Biobank (Petersen et al., 2013). This will confirm the wider applicability of the QCD framework, to promote its utilization by others in the medical imaging research community.

### 4.3 Clinical impact

Assuming equal variation in the automatic and the human estimates, the reported 95% CI range here in the Bland-Altman plot translates to a small standard deviation of 1.3% for the mean percentage error, and 0.9% for the mean absolute percentage error. Such high agreement in estimated T1 values between the automated and the manual segmentations implies that the automated segmentation can minimize the burden of manual processing and improve time efficiency in both real-time clinical practice and large-scale research, to consistently extract T1-related clinical parameters at the level of human operators. For real-time clinical application, the framework could be integrated into MRI scanners to generate an immediate segmentation after an image is acquired for instant availability for interpretation. For large-scale clinical research and trials, the automation of segmentation can reliably process tens of thousands of datasets, saving labor-intensive processing and costs for processing large-scale imaging databases.

Across all these applications, there is an added benefit from the highly accurate quality prediction, which can reduce the effort to manually screen the data for any suboptimal results. Future work is pending to establish relevant quality thresholds, to further improve reliability of the automated segmentation to identify error-prone datasets in large-scale clinical data. This will help improve robustness to detect and interpret outlier data without excessive workload on human observers to manually score data quality. With improved quality of clinical parameters and reduction in errors, it may reduce sample sizes required for expensive clinical studies or trials, saving resources.

### 4.4 Conclusion

The QCD framework for automated quality prediction improves the accuracy and the robustness of the segmentation. The quality control exploits differences among models to predict each segmentation quality, without the need for manual contour ground truth. The predicted quality score can also be used for binary classification of segmentation quality. The selection of the most optimal segmentation is performed on-the-fly using the quality prediction, and significantly improves the accuracy above any individual network or their combinations. The proposed segmentation agreement visualization provides a simple tool to monitor the quality control process. The validation on the cardiac magnetic resonance T1 mapping data shows wider adaptability of the framework. The automated estimates of T1 relaxation times showed near-perfect agreement (r=0.987,p<.0005; mean absolute error (MAE) of 11.3ms) with the manual estimation used in clinical research, with a fast processing speed of 0.39s/image. The use of the QCD framework could lead to real-time parameter extraction in clinical practice and automation of labor-intensive tasks in large-scale clinical research and trials. This can enable clinicians and healthcare personnel to spend more time with patients rather than performing tedious segmentation and quality control tasks.

## CRediT authorship contribution statement

**Evan Hann:** Conceptualization, Methodology, Software, Validation, Formal analysis, Investigation, Resources, Data curation, Writing - original draft, Writing - review & editing, Funding acquisition. **Iulia A. Popescu:** Conceptualization, Writing - original draft, Supervision, Writing - review & editing, Software, Validation, Data curation. **Qiang Zhang:** Conceptualization, Methodology, Software, Validation, Investigation, Writing - original draft, Writing - review & editing, Supervision. **Ricardo A. Gonzales:** Software, Validation, Data curation, Writing - review & editing. **Ahmet Barutçu:** Formal analysis, Investigation, Resources, Data curation, Writing - review & editing. **Stefan Neubauer:** Resources, Supervision, Project administration, Funding acquisition, Writing - review & editing. **Vanessa M. Ferreira:** Conceptualization, Validation, Resources, Writing - original draft, Writing - review & editing, Supervision, Project administration, Funding acquisition. **Stefan K. Piechnik:** Conceptualization, Methodology, Validation, Formal analysis, Resources, Data curation, Writing - original draft, Writing - review & editing, Supervision, Project administration, Funding acquisition.

## Declaration of Competing Interest

The authors declare the following financial interests/personal relationships which may be considered as potential competing interests:

Authors declare that a patent is filed for the methods used in this submitted work.

## Figures and Tables

**Table A.1:** Multiple linear regression coefficients for the DSC predictors are shown. Each row represents a specific prediction target DSC (manual ground truth, target segmentation) with coefficients for the corresponding DSCs and an intercept. Comb. denotes ”Combined Model”.

Prediction Target	DSC(target, U-net 7)	DSC(target, U-net 11)	DSC(target, U-net 15)	DSC(target, U-net 19)	DSC(target, U-net 23)	DSC(target, U-net 27)	DSC(target, Comb. 1)	DSC(target, Comb. 2)	DSC(target, Comb. 3)	DSC(target, Comb. 4)	DSC(target, Comb. 5)	DSC(target, Comb. 6)	Intercept
**DSC(Manual, U-net 7)**	0.00	0.04	0.11	0.00	0.26	0.06	0.00	0.00	0.25	0.20	0.00	0.01	0.00
**DSC(Manual, U-net 11)**	0.00	0.00	0.26	0.00	0.19	0.07	0.00	0.00	0.29	0.10	0.00	0.01	0.00
**DSC(Manual, U-net 15)**	0.00	0.18	0.00	0.00	0.04	0.03	0.06	0.02	0.28	0.30	0.01	0.00	0.00
**DSC(Manual, U-net 19)**	0.01	0.10	0.12	0.00	0.21	0.06	0.05	0.00	0.26	0.10	0.00	0.01	0.00
**DSC(Manual, U-net 23)**	0.01	0.12	0.00	0.34	0.00	0.00	0.07	0.00	0.14	0.20	0.03	0.01	0.00
**DSC(Manual, U-net 27)**	0.00	0.12	0.01	0.28	0.01	0.00	0.11	0.00	0.10	0.00	0.25	0.01	0.04
**DSC(Manual, Comb. 1)**	0.00	0.00	0.06	0.31	0.02	0.00	0.00	0.00	0.33	0.06	0.13	0.01	0.00
**DSC(Manual, Comb. 2)**	0.01	0.00	0.00	0.53	0.07	0.01	0.11	0.00	0.00	0.01	0.17	0.01	0.00
**DSC(Manual, Comb. 3)**	0.01	0.02	0.00	0.56	0.05	0.00	0.11	0.00	0.00	0.00	0.16	0.02	0.00
**DSC(Manual, Comb. 4)**	0.00	0.06	0.01	0.29	0.01	0.00	0.11	0.31	0.00	0.00	0.14	0.00	0.00
**DSC(Manual, Comb. 5)**	0.01	0.00	0.01	0.00	0.17	0.00	0.02	0.36	0.36	0.00	0.00	0.00	0.00
**DSC(Manual, Comb. 6)**	0.00	0.13	0.00	0.00	0.00	0.00	0.00	0.27	0.32	0.20	0.03	0.00	0.00

**Fig. B.1:**
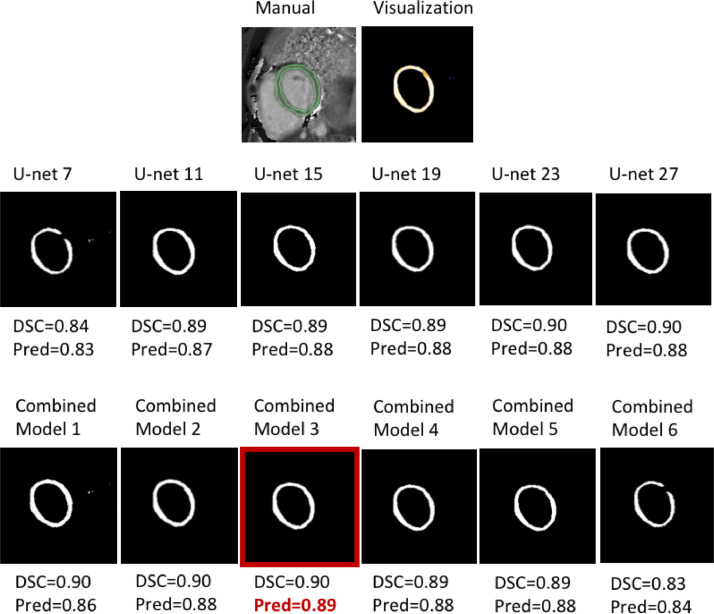
Extended example of Fig. 2A-D showing high agreement among candidate segmentations for a good quality T1 map. The top left image shows the manual segmentation and the top right image shows the visualization of the candidate segmentations. The rest shows the candidate segmentations from U-net 7 to U-net 27, and the combined segmentations (Model 1 - Model 6). All the candidate segmentations consistently obtained high DSCs of ≥0.83, as the good quality T1 map was easy to segment. Combined Model 3 (in the red box) achieved the highest predicted DSC (0.89), thus its segmentation was selected as the final output by the QCD framework. (For interpretation of the references to colour in this figure legend, the reader is referred to the web version of this article.)

**Fig. B.2:**
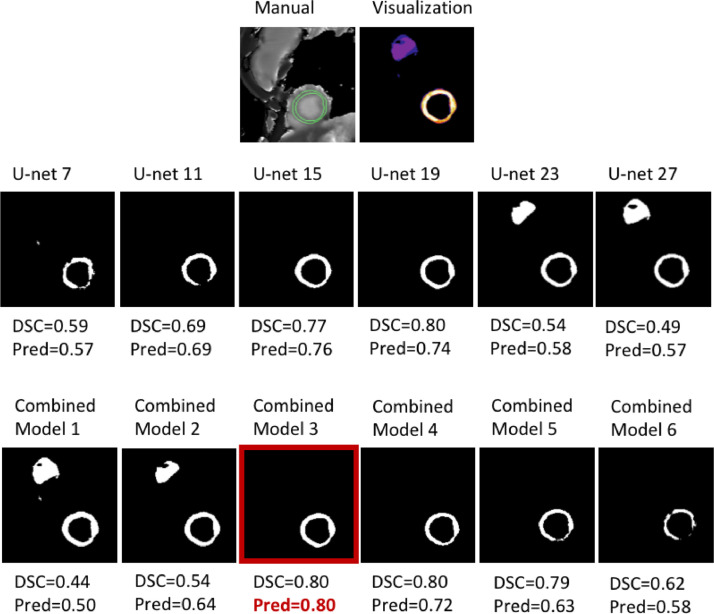
Extended example of Fig. 2M-P showing poor agreement in the candidate segmentation failures of a T1-map affected by an extracardiac structure (breast implant). The top left image shows the manual segmentation and the top right image shows the visualization of the candidate segmentations. The rest shows the candidate segmentations from U-net 7 to U-net 27, and the combined segmentations (Model 1 - Model 6). When the candidate U-nets failed, they appeared to fail differently, as demonstrated by U-net 7, 11, 23, and 27, obtaining a DSC of 0.59, 0.68, 0.54, and 0.49, respectively. U-nets 7 and 11 failed to form an annulus-like myocardial mask, whereas U-nets 23 and 27 falsely identified the breast implant as part of the myocardium. Despite the difficulty, U-nets 15 and 19, and Combined Models 3 and 4 successfully segmented the myocardium, with DSCs ≥0.77. This illustrates the importance of including a diverse set of candidate models. Combined Model 3 (in the red box) achieved the highest predicted DSC (0.80), thus its segmentation was selected as the final output by the QCD framework. (For interpretation of the references to colour in this figure legend, the reader is referred to the web version of this article.)

**Table D.1:** Segmentation performance for candidate models and the QCD in mean Dice similarity coefficient (DSC). Comb. denotes ”Combined Model”.

Mean DSC (SD)			
Model	Myocardium	Endocardium	Epicardium
U-net 7	0.5900 (0.2302)	0.5028 (0.2406)	0.5908 (0.2420)
U-net 11	0.7533 (0.1697)	0.8731 (0.1582)	0.8914 (0.1579)
U-net 15	0.7952 (0.1341)	**0.9165** (0.1267)	0.9389 (0.1215)
U-net 19	0.7795 (0.1578)	0.8895 (0.1695)	0.9291 (0.1408)
U-net 23	0.7943 (0.1370)	0.9122 (0.1337)	0.9371 (0.1233)
U-net 27	0.7838 (0.1375)	0.9051 (0.1357)	0.9254 (0.1300)
Comb. 1	0.7510 (0.1264)	0.8495 (0.1464)	0.8732 (0.1273)
Comb. 2	0.8033 (0.1333)	0.9137 (0.1293)	0.9362 (0.1223)
Comb. 3	0.8026 (0.1362)	0.9126 (0.1380)	0.9377 (0.1278)
Comb. 4	0.7899 (0.1439)	0.9029 (0.1583)	0.9323 (0.1372)
Comb. 5	0.7364 (0.1883)	0.8804 (0.1743)	0.9023 (0.1672)
Comb. 6	0.5888 (0.2544)	0.5131 (0.2566)	0.6116 (0.2616)
**QCD**	**0.8039** (0.1355)	0.9162 (0.1303)	**0.9403** (0.1194)

**Table D.2:** DSC prediction performance in mean absolute error (MAE) and Pearson correlation coefficient (r). All r had p<.0005.

	Myocardium		Endocardium		Epicardium	
Model	MAE	*r*	MAE	*r*	MAE	*r*
U-net 7	0.0354	0.98	0.0175	0.99	0.0119	0.99
U-net 11	0.0469	0.93	0.0252	0.98	0.0181	0.99
U-net 15	0.0538	0.86	0.0323	0.81	0.0249	0.92
U-net 19	0.0601	0.88	0.0296	0.97	0.0215	0.95
U-net 23	0.0539	0.86	0.0289	0.94	0.0158	0.98
U-net 27	0.0563	0.87	0.0273	0.94	0.0211	0.97
Comb. 1	0.0547	0.81	0.0365	0.92	0.0197	0.98
Comb. 2	0.0579	0.86	0.0286	0.92	0.0218	0.98
Comb. 3	0.0534	0.85	0.0247	0.96	0.0187	0.97
Comb. 4	0.0519	0.87	0.0280	0.97	0.0189	0.96
Comb. 5	0.0478	0.95	0.0277	0.98	0.0184	0.99
Comb. 6	0.0375	0.98	0.0231	0.99	0.0146	0.99
QCD	0.0620	0.87	0.0298	0.92	0.0191	0.98

**Fig. D.1:**
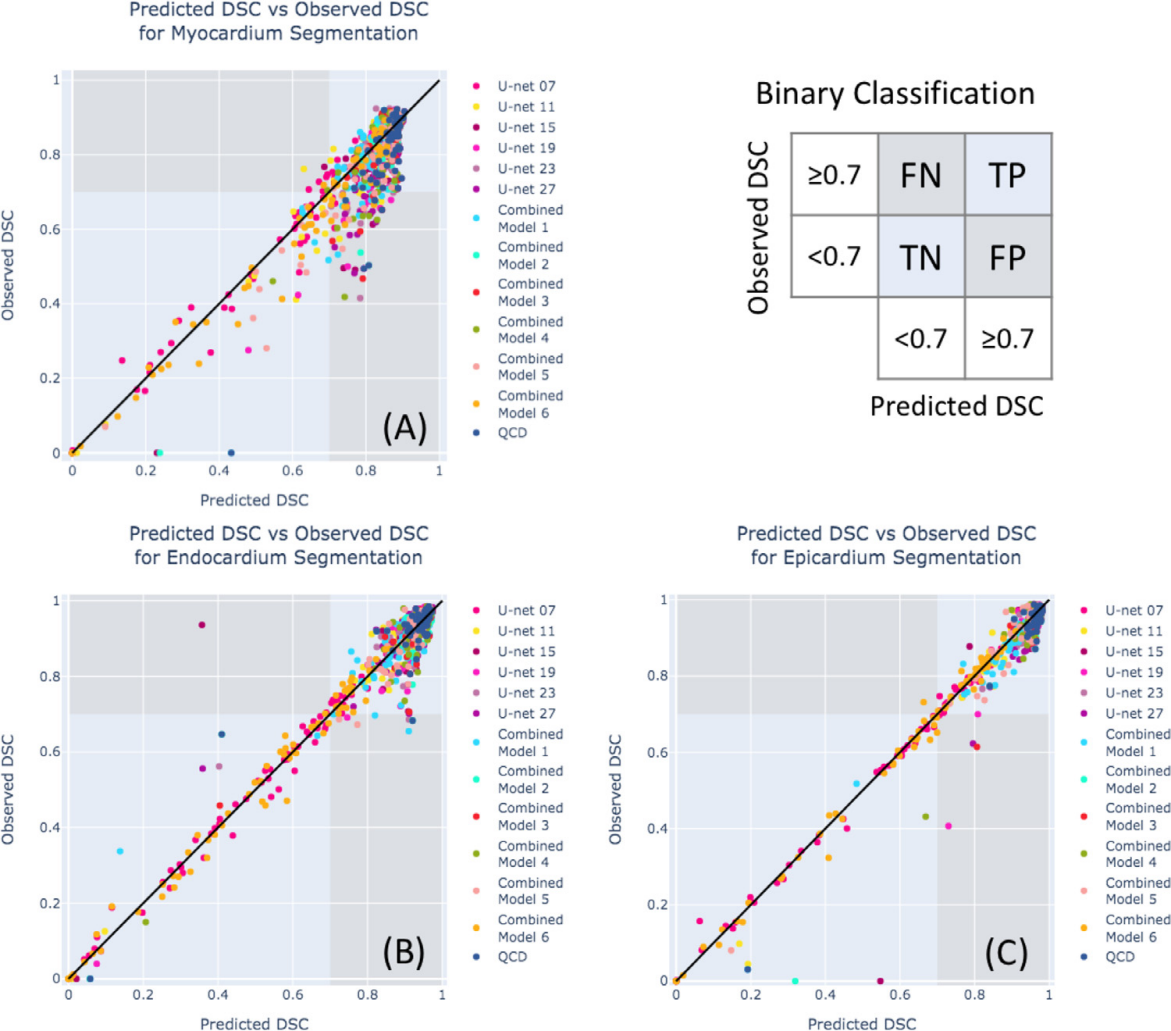
Scatter plots for predicted DSC (x-axis) versus observed DSC (y-axis) for the myocardium (A), the endocardium (B), and the epicardium (C).

## Appendix B Examples of candidate segmentations for good quality and bad quality T1 maps

Two sets of example candidate segmentations are shown for an easy, good-quality T1 map (Fig. B.1) and a difficult T1 map affected by an extracardiac structure (breast implant) (Fig. B.2). These examples illustrate the agreement among the candidate segmentations under 2 different scenarios. With a good quality T1 map, Fig. B.1 shows high agreement among the candidates with high DSCs of ≥0.83. In contrast, Fig. B.2 shows high disagreement, as some of the candidate segmentations failed differently, including falsely identifying the breast implant as the myocardium, and failures to segment the whole myocardium. These demonstrate that segmentation differences from a diverse set of candidates can be exploited to estimate segmentation quality.

## Appendix D Additional results: sunnybrook cardiac dataset

The Sunnybrook cardiac MRI short-axis SSPF cine dataset of 45 subjects was split into 38 training subjects and 7 testing subjects, with one to two subjects chosen from each pathology or healthy group to be the testing data (Radau et al., 2009). Since both the endocardium and the epicardium were contoured only at end-diastole (no epicardial contours at the end-systole), only the end-diastolic images were used for training (355 images) and testing (65 images). In addition, the ground truth myocardial segmentation masks were derived by subtracting the endocardial masks from the epicardial masks.

As the Sunnybrook dataset was relatively small in the context of deep learning applications, data augmentation was performed for the training data by randomly rotating the images and masks within ±10 degrees. 6 U-nets were independently trained on the augmented data for up to 240 epochs to perform endocardial and myocardial segmentation. The epicardial masks were calculated post-hoc by adding the endocardial masks and the myocardial masks. 6 combined segmentations were generated in addition to 6 candidate segmentations generated by the U-nets. Linear regression models were trained to exploit segmentation agreement among candidates to predict segmentation quality. The final segmentation was selected based on the best predicted myocardial segmentation DSC.

All the candidate segmentation models and the QCD were evaluated for the segmentation performance. Table D.1 shows the mean Dice similarity coefficients (DSC) for segmentation of the myocardium, the endocardium, and the epicardium. The QCD framework obtained the highest mean DSC for the myocardium at 0.8039 and for the epicardium at 0.9403, outperforming all the candidate segmentation models. U-net 15 obtained the highest mean DSC for the endocardium at 0.9165, closely followed by the QCD at 0.9162. Although similar segmentation performance has been reported by the prior art (Huang, Liu, Lee, Venkatesh, Li, Teo, 2009, Wijnhout, Hendriksen, Assen, Der, 2009, Jolly, 2009, Lu, Radau, Connelly, Dick, Wright, 2009), the QCD framework achieved the best segmentation performance with an added layer of the automated quality assurance.

For evaluation of the quality control component, mean absolute errors (MAE) and Pearson correlation coefficients (r) between the predicted DSCs and the observed DSCs are reported in Table D.2. All of the DSC predictions achieved low MAEs within 0.0620, and high Pearson r above 0.80. These results demonstrate the high accuracy for the DSC prediction.

Fig. D.1 shows 3 scatter plots of the predicted DSC versus the observed DSC for the myocardium (Fig. D.1A), the endocardium (Fig. D.1B), and the epicardium (Fig. D.1C), where most of the data points cluster along the identity line, indicating high agreement between the ground truth and the prediction. Furthermore, the DSC prediction was extended to classify ‘good’ and ‘bad’ segmentation with a threshold of 0.7. All the candidate models together achieved a classification accuracy of 89%, 99%, and 99% for the myocardium, the endocardium, and the epicardium, respectively. For the QCD framework, high accuracies were also achieved for the myocardium (92%), the endocardium (98%), and the epicardium (100%). These results show that the quality prediction can be extended to achieve accurate classification of segmentation quality also in cardiac cine applications.
